# Essential palatal myoclonus with spontaneous resolution: a rare case report

**DOI:** 10.1097/MS9.0000000000003924

**Published:** 2025-09-17

**Authors:** Prakriti Lamichhane, Dirishya Bishowkarma, Saral Lamichhane, Shanti Sharma, Shankar Rimal

**Affiliations:** a Department of Internal Medicine, Pokhara Hospital and Research Centre, Kaski, Nepal; b Department of General Medicine, Armala Primary Health Care, Kaski, Nepal; c Department of Internal Medicine, Woodhull Medical Center, New York, USA; d Department of Internal Medicine, Kalika Nagar Hospital, Chitwan, Nepal; e Department of Emergency Medicine, HI-CARE Hospital, Kathmandu, Nepal

**Keywords:** essential palatal myoclonus, palatal tremor, rhythmic palatal movement

## Abstract

**Introduction and Importance:**

Palatal myoclonus is a rare movement disorder characterized by involuntary, jerky movements of the soft palate and palatal musculature. It can be broadly divided into two types: essential palatal myoclonus (EPM) and symptomatic palatal myoclonus (SPM). In this case report, we report a rare case of EPM in a pediatric patient, which resolved on its own during follow-up.

**Case Presentation:**

An 8-year-old male presented with upper respiratory symptoms, with further examinations and investigations leading to the diagnosis of EPM. The patient had no accompanying neurological symptoms, and brain imaging revealed no distinct lesions. The presentation was incidental and was resolved spontaneously within 1 year.

**Clinical Discussion:**

This case reports a rare occurrence of EPM in a pediatric patient. Careful clinical assessment is required to differentiate SPM, and brain imaging also helps to rule out any brain pathologies leading to palatal myoclonus.

**Conclusion:**

This case report contributes to scant pediatric literature on EPM and emphasizes the need for clinicians to understand the benign nature in order to prevent unnecessary investigations and treatment.

## Introduction

Palatal myoclonus, also referred to as palatal nystagmus or palatal tremor, is a rare condition^[1]^. It presents as involuntary, rapid, rhythmic, jerky movements of the peritubular muscles and soft palate with an audible click^[2]^.

There are two known forms of palatal myoclonus: essential palatal myoclonus (EPM) and symptomatic palatal myoclonus (SPM). A brainstem lesion, usually affecting the upper cerebellar peduncle (the dentate–rubro–olivary triangle of Guillain–Mollaret), causes the disease process in SPM^[3]^. EPM is the condition when no distinct lesion is found and is linked to the tensor veli palatini’s rhythmic activation; often frequently detected by hearing a click that stops with sleep^[3]^. With varying degrees of success, a number of therapy approaches have been tested, including anxiolytics, anticonvulsants, botulinum toxin, and surgery^[4]^. This case report has been reported in line with the SCARE Criteria [Sohrabi C, Mathew G, Maria N, Kerwan A, Franchi T, Agha RA. The SCARE 2023 guideline: updating consensus Surgical CAse REport (SCARE) guidelines. Int J Surg Lond Engl. 2023;109(5):1136]. It has also been reported in line with the TITAN guideline^[5]^.

**HIGHLIGHTS**

- To present a case of EPM in a child with spontaneous resolution.
- To discuss diagnostic and therapeutic challenges.
- This case report shares the importance of clinical differentiation of types of myoclonus, thereby decreasing unnecessary investigations.
- This case report highlights how crucial is close monitoring and reassurance for the management of EPM.

## Case presentation

An 8-year-old male was presented to our hospital with a history of cough and runny nose for 5 days. Cough was dry in nature, which was more during nighttime and associated with throat irritation. There was also a history of fever for 5 days, gradual in onset, maximum temperature was recorded as 100°F without chills and rigors. He had no history of hearing loss, vertigo, ear discharge, or otalgia. He denied having headache, head trauma, or weakness in his body. There was no history of nasal regurgitation, dysphagia, shortness of breath, or nasal speech. There were no significant past medical, surgical, or family history. Vaccinations were up to date.

**Figure 1.**
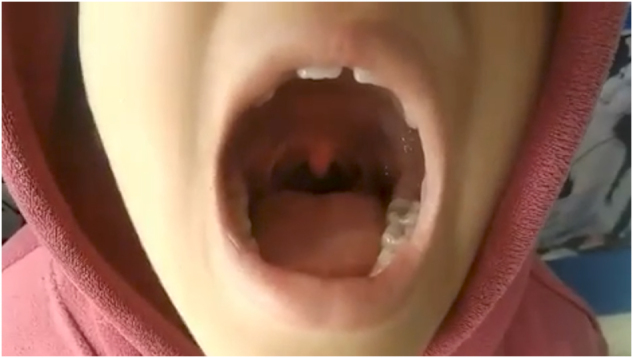
Oral Cavity Examination of Palatal myoclonus representative of rhythmic movement of both soft palate and uvula with audible click sound.

On examination of the oral cavity, there was spontaneous, rhythmic movement of the uvula and soft palate on both sides (Fig. 1, Supplemental Digital Content Video 1, available at, http://links.lww.com/MS9/A946). An auditory click sound was heard only when the mouth was opened. Detailed ophthalmological, neurological, nasal, ear and head and neck examinations were done, which revealed no other abnormalities. Routine blood investigations were done, which were within normal limits. MRI brain was done to rule out any brain lesions, which were normal. A psychiatry consultation was done, which was unremarkable. Patient reported no distress and was unaware of the movement before. He was managed symptomatically for fever and cough. Since there were no significant physical examination findings and investigations were normal, he was later diagnosed with EPM.

After explaining the diagnosis and management options, the patient’s mother denied any treatment as the patient did not report any distress. So, the patient and his mother were reassured, and the patient was monitored with regular follow-up. The audible click disappeared at the 6-month follow-up, although the rhythmic movement of uvula and soft palate was persistent. No intervention was carried out, and patient and his mother were reassured. On 1-year follow-up symptoms were resolved completely without intervention.

## Timeline

Day 1 (8 December 2023): Symptoms appeared

- Fever (maximum temperature of 100°F, without chills or rigors) started, along with a dry cough and runny nose.

Day 5 (13 December 2023): Presented to the hospital

- Five days of fever and cough and running nose.

No respiratory, neurological, or ear warning signs; the cough gets worse at night; and the throat gets irritated.

- General examination: Normal.
- Systemic examination:

Neurological, ophthalmological, nasal, ear, and head and neck examinations: Normal.

Oral cavity examination: A clicking sound that is audible only when the mouth is opened; rhythmic, spontaneous palatal and uvular movement observed.

Day 6 (14 December 2023): Investigations: Regular blood examinations: Normal

Day 7 (15 December 2023): Brain MRI: Normal

EPM was diagnosed; no secondary etiology was found.Management: Medication for fever and cough symptoms; no palatal myoclonus medication was started at the mother’s request.

Six months follow-up: Audible click disappeared, rhythmic movement of uvula and soft palate present. Reassured.

One-year follow-up: Completely resolved on its own.

## Discussion

Palatal myoclonus is a repetitive, involuntary, jerky movement of the soft palate and pterygopalatine arches and rarely involves the facial muscles^[6]^. There are two subtypes of palatal myoclonus: (1) essential and (2) symptomatic^[6]^.

EPM is characterized by involuntary, continuous, rhythmic movement of the muscles of soft palate and uvula without an organic lesion. It usually involves the tensor veli palatini muscles and is often associated with an audible click^[6,7]^. Some people describe ear clicking as banging, ticking, popping, crunching, or crackling noises^[8]^. It could be both unilateral and bilateral^[6]^.

One study shows that nine cases of essential palatal tremor had objective tinnitus reported during childhood^[9]^. In those children, there was no known etiology, and they had a variable clinical course^[9]^. In four cases, tinnitus was absent while sleeping, which is similar like our case, and some were able to stop tinnitus by doing a valsalva technique, unlike our case^[9]^. It could be confused with fasciculations due to upper motor neuron disease, but in our case, there were no associated neurological abnormalities and focal neurological deficits. There was no history of triggers for myoclonus, and the movement is present throughout the day, while it is absent during the sleep, indicating EPM. Brain imaging and electromyography are important investigation work up for this case^[4]^. In case of EPM, there are no demonstrable lesions seen in the brainstem or cerebellum, which is similar in our case^[4]^. Due to resource limitations and the fact that EMG often does not change management when MRI is normal and the clinical features are suggestive of EPM, we did not take EMG into consideration. However, EMG is helpful to differentiate muscles involved, especially in complex cases^[4]^.

In contrast to the EPM, SPM is a condition secondary to pathology of brain^[10]^. It is usually associated with tinnitus, abnormal eyeball movement, vertigo, and weakness of body part^[11]^. Myoclonus is present during sleep, and ocular involvement is frequently observed^[10]^. Pontine stroke is one of the most important cause of SPM, of which pontine hemorrhages are more likely than pontine infarcts^[11]^. Hypertrophic degeneration of the inferior olivary nucleus is the pathological reason most frequently seen in patients with SPM^[11]^.

There are multiple differential diagnoses for the palatal myoclonus, such as multiple sclerosis, trauma, tumor, arteriovenous malformation and venous hums, middle ear myoclonus, eustachian tube dysfunction, and psychogenic EPM^[8]^. In case of multiple sclerosis, there should be asymmetrical limb weakness, paresthesia, urinary incontinence, and ophthalmoplegia, which were all absent in our case^[8]^. There was no history of trauma. Brain MRI excluded intracranial lesions and arteriovenous malformation. A normal tympanic membrane ruled out middle ear myoclonus, in which the tympanic membrane appears as an oscillating structure^[8]^. Aural fullness and muffled hearing are not present in this case, which also excludes eustachian tube dysfunction^[11]^. No history of emotional stress or previous history of trauma and unremarkable psychiatric consultation ruled out psychogenic EPM^[8]^. Our findings showed that our patient has been suffering from an unclear etiology of palatal myoclonus, hence diagnosed as EPM.

EPM has been treated with a variety of medications^[6]^. Conservative treatment with few maneuvers has been applied, like pressing over the mastoid or adjusting the neck to a certain posture, with limited benefits^[8]^. The mechanism of these maneuvers works by changing the tone of the muscles involved and by changing pressure of the ear canal^[8]^.

Medications include anticonvulsants such as sodium valproate, carbamazepine, and clonazepam^[6]^. Clonazepam and flunarazine are more effective in the treatment of essential myoclonus compared to sodium valproate and subcutaneous sumatriptan^[6]^. Although cannabis has also been explored, the most effective way to manage symptoms for up to 3 months has been with a botulinum toxin injection^[2]^. The clinical course of objective tinnitus and palatal tremor reported in children is usually benign, and some patients experience a resolution of their symptoms^[9]^. In our case, the patient’s mother denied any treatment as the patient did not report distress, hence was reassured and later resolved spontaneously within 1 year. There are only a few reported cases of essential myoclonus^[6]^. We hope that our case will help medical professionals who are dealing with diagnostic or therapeutic challenges due to the rarity and potential to improve clinical understanding of different types of palatal myoclonus.

## Conclusion

EPM is a rare, benign, and self-limiting condition. It is important to recognize the clinical symptoms of EPM and rule out neurological symptoms in order to differentiate from SPM, thereby reducing unnecessary investigations and treatments. This case demonstrates how crucial is clinical monitoring and reassurance in the management of EPM.

## Data Availability

Not applicable.
